# Supplementary material for: Reducing nonradiative recombination for highly efficient inverted perovskite solar cells via a synergistic bimolecular interface
Source: Nat Commun. 2024 Jul 4;15:5607. doi: 10.1038/s41467-024-50019-3 (PMC11224317; doi:10.1038/s41467-024-50019-3)
Supplement: Supplementary file 1 — Supplementary Information [file 41467_2024_50019_MOESM1_ESM.pdf]

Supporting information

Reducing nonradiative recombination for highly efficient inverted perovskite solar cells via a synergistic bimolecular interface

Shaobing Xiong[1,2†], Fuyu Tian[3†], Feng Wang[4†], Aiping Cao[1], Zeng Chen[5], Sheng Jiang[1], Di Li[1], Bin Xu[1], Hongbo Wu[6], Yefan Zhang[7], Hongwei Qiao[1], Zaifei Ma[6], Jianxin Tang[7], Haiming Zhu[5], Yefeng Yao[1], Xianjie Liu[8], Lijun Zhang[3]*, Zhenrong Sun[9], Mats Fahlman[8], Junhao Chu[2], Feng Gao[4]*, Qinye Bao[1,2,10]*

[1]*School of Physics and Electronic Science, East China Normal University, Shanghai 200241, China*

[2]*Shanghai Frontiers Science Research Base of Intelligent Optoelectronics and Perception, Institute of Optoelectronics, Fudan University, Shanghai 200433, China*

[3]*State Key Laboratory of Integrated Optoelectronics, Key Laboratory of Automobile Materials of MOE, International Center of Computational Method and Software, School of Materials Science and Engineering, Jilin University, Changchun 130012, China*

[4]*Department of Physics, Chemistry and Biology, Linköping University, Linköping 58183, Sweden*

[5]*Chemistry of High-Performance and Novel Materials, Department of Chemistry, Zhejiang University, Hangzhou 310027, China*

[6]*Center for Advanced Low-Dimension Materials, Donghua University, Shanghai 201620, China*

[7]*Institute of Functional Nano & Soft Materials (FUNSOM), Soochow University, Suzhou 215123, China*

[8]*Laboratory of Organic Electronics, Linköping University, Norrköping 60174, Sweden*

[9]*State Key Laboratory of Precision Spectroscopy, East China Normal University, Shanghai 200241, China*

[10]*Collaborative Innovation Center of Extreme Optics, Shanxi University, Taiyuan, Shanxi 030006, China*

[†]*These authors contributed equally: Shaobing Xiong, Fuyu Tian, Feng Wang*

*Corresponding authors.

E-mail: lijun_zhang@jlu.edu.cn (L. Z.); feng.gao@liu.se (F. G.); qybao@clpm.ecnu.edu.cn (Q. B.)

Supplementary Fig. 1. Top-view SEM images of (a) control, (b) MPA- and (c) SBI-modified perovskite films.

Supplementary Fig. 2. XRD patterns of control, MPA- and SBI-modified perovskite films.

Supplementary Fig. 3. UV-Vis spectra of control, MPA- and SBI-modified perovskite films.

Supplementary Fig. 4. Water contact angle of (a) control, (b) MPA- and (c) SBI-modified perovskite films.

Supplementary Fig. 5. XPS measurement of perovskite films with MPA. XPS (a) Pb 4*f*, (b) N 1*s*, (c) O 1*s*, (d) I 3*d*, (e) P 2*p* and (f) C 1*s* core level spectra of control and MPA-modified perovskite with different concentrations. Here, MPA3, MPA5 and MPA7 refer to MPA with concentrations of 3, 5 and 7 mg ml^{-1}, respectively.

Supplementary Fig. 6. pH test of ethanol solvent with and without MPA.

Supplementary Fig. 7. XPS (a) O 1*s* and (b) P 2*p* core level spectra of pristine MPA.

Supplementary Fig. 8. FTIR spectra of MPA, perovskite, MPA-modified perovskite films. The fingerprint regions highlight (a) P-OH, P-O, P=O stretching and (b) NH stretching.

Supplementary Fig. 9. XPS measurement of perovskite films with SBI. XPS (a) C 1*s*, (b) I 3*d* and (c) P 2*p* core level spectra of control, MPA- and SBI-modified perovskite films.

Supplementary Fig. 10. UPS spectra of secondary electron cutoff region and valence band region of control and MPA-modified perovskite films with different concentrations of 3, 5 and 7 mg ml^{-1}.

Supplementary Fig. 11. Topography images of control, MPA-, SBI- and PEAI-modified perovskite films.

Supplementary Fig. 12. Dark *J-V* curves of electron-only devices with and without SBI modification.

Supplementary Fig. 13. Impact of SBI on charge carrier dynamics. Two-dimensional (2D) pseudo-color plots of the fs-TA results for (a) control and (b) SBI-

modified perovskite films. fs-TA spectra at selected pump-probe delay time of (c) control and (d) SBI-modified perovskite films.

Supplementary Fig. 14. The GSB decays at 770 nm of control and SBI-modified perovskite films.

Supplementary Fig. 15. fs-TA spectra at selected pump-probe delay time of (a) control and (b) SBI-modified perovskite films with ETL.

Supplementary Fig. 16. *J-V* curves of control and SBI-based PSCs with concentrations of 1, 3, 5 and 7 mg ml^{-1}. An optimal MPA concentration of 5 mg ml^{-1} is obtained.

Supplementary Fig. 17. *J-V* curves of control and SBI-based PSCs with MPA concentration of 5 mg ml^{-1} and PEAI concentrations of 0.5, 1, 3 and 5 mg ml^{-1}. An optimal PEAI concentration of 1 mg ml^{-1} is obtained.

Supplementary Fig. 18. Device performance certification report by Shanghai Institute of Microsystem and Information Technology (SIMIT), Chinese Academy of Sciences. All elements in Supplementary Fig. 18 have received written approval from the copyright holder.

Supplementary Fig. 19. *J-V* curves of control, MPA- and SBI-based PSCs under forward scan (FS) and reverse scan (RS).

Supplementary Fig. 20. *J-V* curves of PSCs with MPA/PEAI and PEAI/MPA modification.

Supplementary Figure 21. *J-V* curves of control, PEAI- and MPA-based PSCs.

Supplementary Fig. 22. Statistics of photovoltaic parameters. (a) V_{oc}, (b) J_{sc}, (c) FF and (d) PCE obtained from 25 control, MPA- and SBI-based PSCs.

Supplementary Fig. 23. *J-V* curves of control, MPA- and SBI-based PSCs using MA-free perovskite $Cs_{0.05}FA_{0.95}Pb(I_{0.95}Br_{0.05})_3$.

Supplementary Fig. 24. *C-V* curves of control, MPA- and SBI-based PSCs.

Supplementary Fig. 25. TPV decays of control, MPA- and SBI-based PSCs.

Supplementary Fig. 26. Dark *J-V* curves of control, MPA- and SBI-based PSCs.

Supplementary Fig. 27. Light intensity dependent V_{oc} of control, MPA- and SBI-based PSCs.

Supplementary Fig. 28. Normalized PCE evolution of control and SBI-based PSCs storage in ambient air at 303 K.

Supplementary Fig. 29. Density of states (DOS) plot of (a) FAI-terminated and (b) PbI-terminated perovskite (001) surface without iodine vacancy.

Supplementary Fig. 30. Theoretical analysis of interaction between perovskite and MPA. (a) Optimized structure of MPA treated PbI-terminated perovskite (001) surface containing an iodine vacancy. (b) Calculated electron localization function and (c) density of states (DOS) projected onto elements of PbI-terminated surface with an iodine vacancy before and after MPA treatment.

Supplementary Table 1. The fitted carrier lifetime of control and SBI modified perovskite films with and without ETL obtained from the TRPL spectra (refer to Fig. 3c).

Supplementary Table 2. Photovoltaic parameters extracted from *J-V* curves of control and MPA-based devices with concentrations of 1, 3, 5 and 7 mg ml^{-1} (refer to Supplementary Figure 16).

Supplementary Table 3. Photovoltaic parameters extracted from *J-V* curves of control and MPA-based devices with MPA concentration of 5 mg ml^{-1} and PEAI concentrations of 0.5, 1, 3 and 5 mg ml^{-1} (refer to Supplementary Figure 17).

Supplementary Table 4. Photovoltaic parameters extracted from *J-V* curves of control and SBI-based devices (refer to Fig. 4a, Supplementary Figure 22).

Supplementary Table 5. Photovoltaic parameters extracted from *J-V* curves of control and PEAI (1 mg ml^{-1})-based devices (refer to Supplementary Figure 21).

Supplementary Table 6. Photovoltaic parameters extracted from *J-V* curves of control and SBI-based devices based on MA-free perovskite $Cs_{0.05}FA_{0.95}Pb(I_{0.95}Br_{0.05})_3$ (refer to Supplementary Figure 23).

Supplementary Table 7. $\Delta V_{oc,\ nonrad}$ and V_{oc} of recent works on p-i-n PSCs (refer to Figure 4f).

Supplementary Note 1. Estimated thickness of the MPA and PEAI by the intensity attenuation of Pb 4*f* XPS spectra.

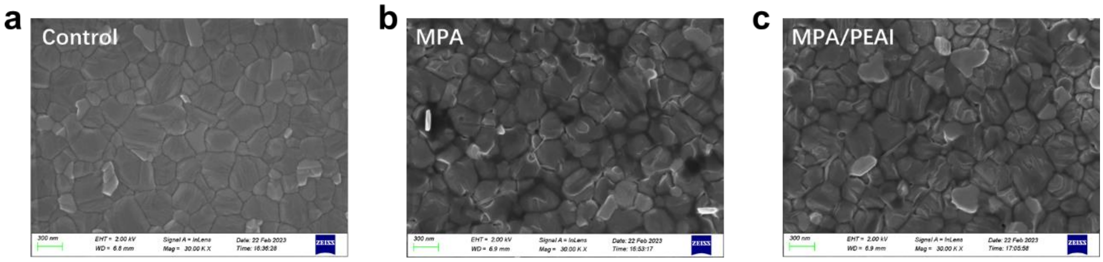

Supplementary Fig. 1. Top-view SEM images of (a) control, (b) MPA- and (c) SBI-modified perovskite films.

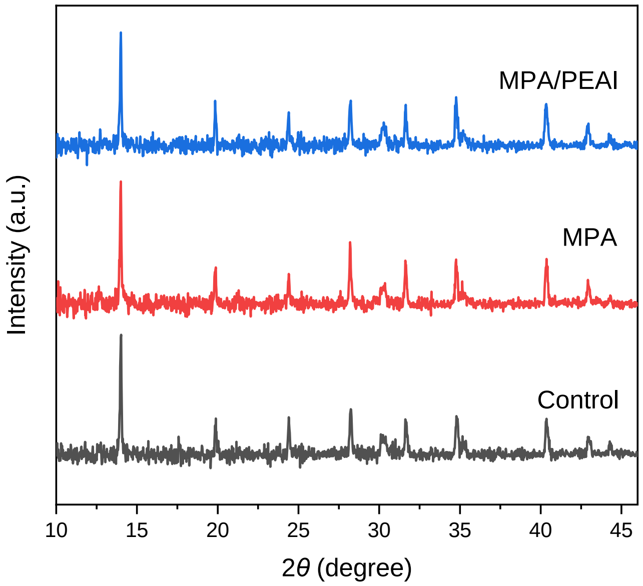

Supplementary Fig. 2. XRD patterns of control, MPA- and SBI-modified perovskite films.

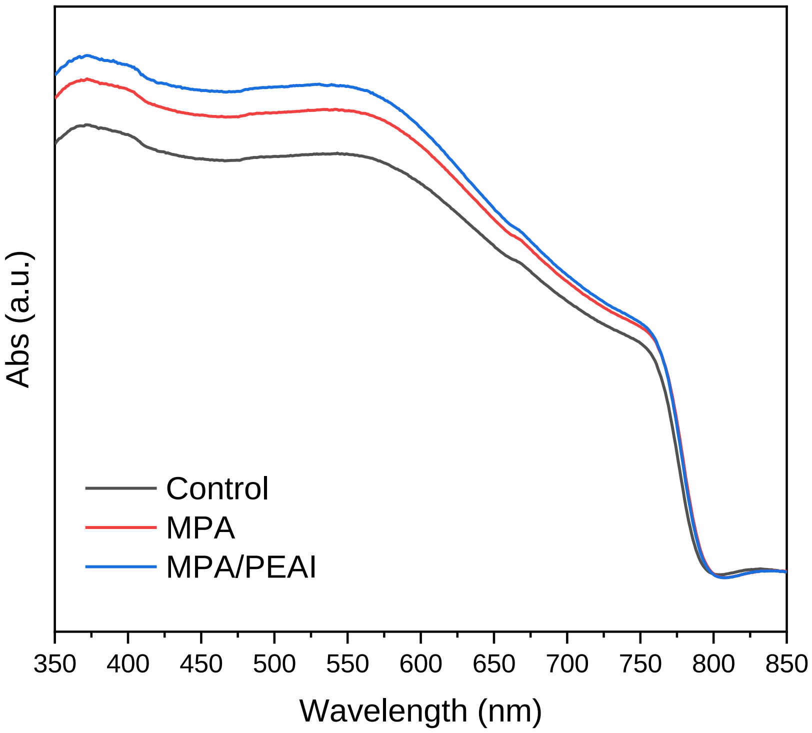

Supplementary Fig. 3. UV-Vis spectra of control, MPA- and SBI-modified perovskite films.

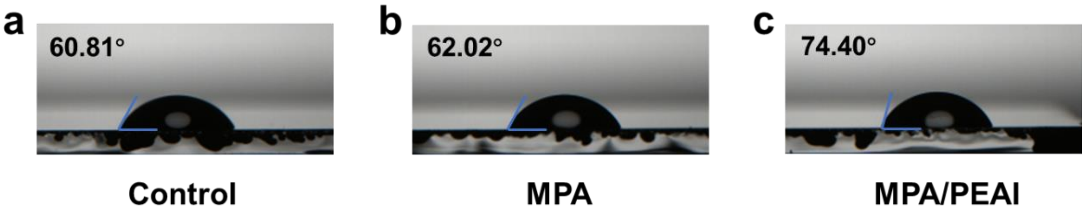

Supplementary Fig. 4. Water contact angle of (a) control, (b) MPA- and (c) SBI-modified perovskite films.

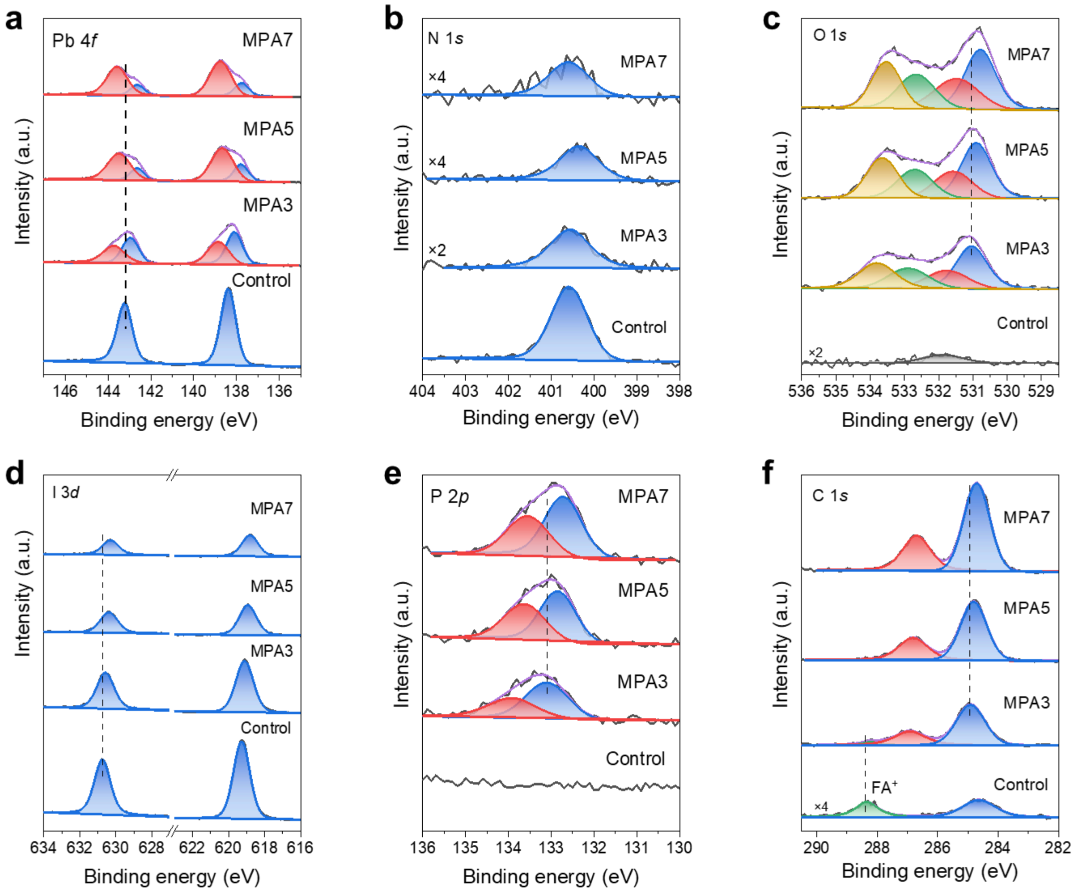

Supplementary Fig. 5. XPS measurement of perovskite films with MPA. XPS (a) Pb 4*f*, (b) N 1*s*, (c) O 1*s*, (d) I 3*d*, (e) P 2*p* and (f) C 1*s* core level spectra of control and MPA-modified perovskite with different concentrations. Here, MPA3, MPA5 and MPA7 refer to MPA with concentrations of 3, 5 and 7 mg ml^{-1}, respectively.

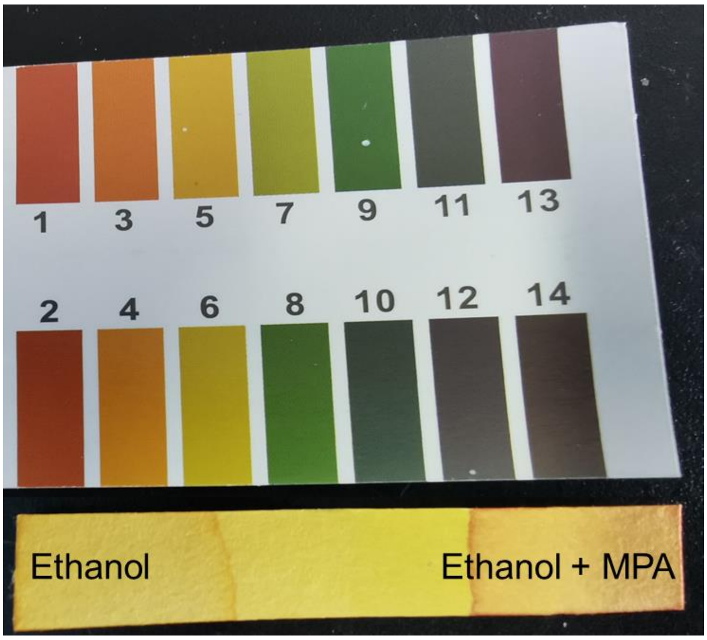

Supplementary Fig. 6. pH test of ethanol solvent with and without MPA.

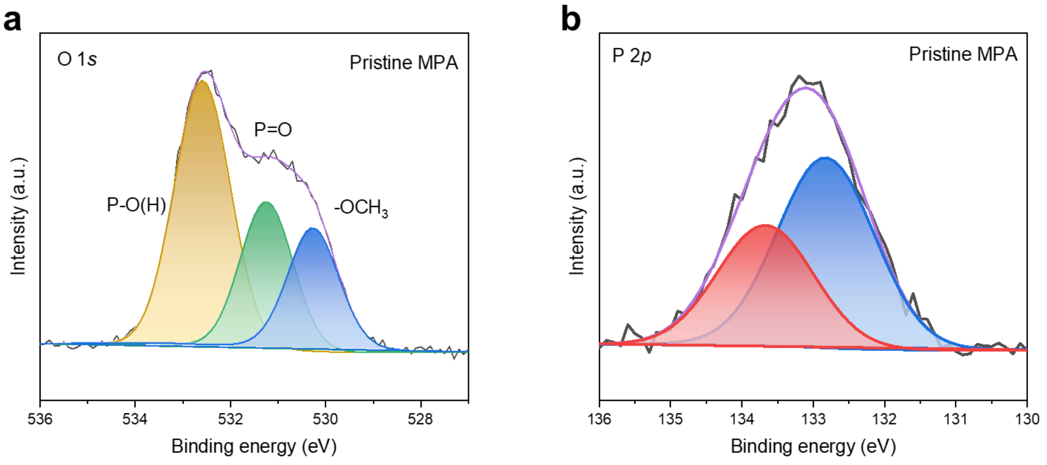

Supplementary Fig. 7. XPS (a) O 1*s* and (b) P 2*p* core level spectra of pristine MPA.

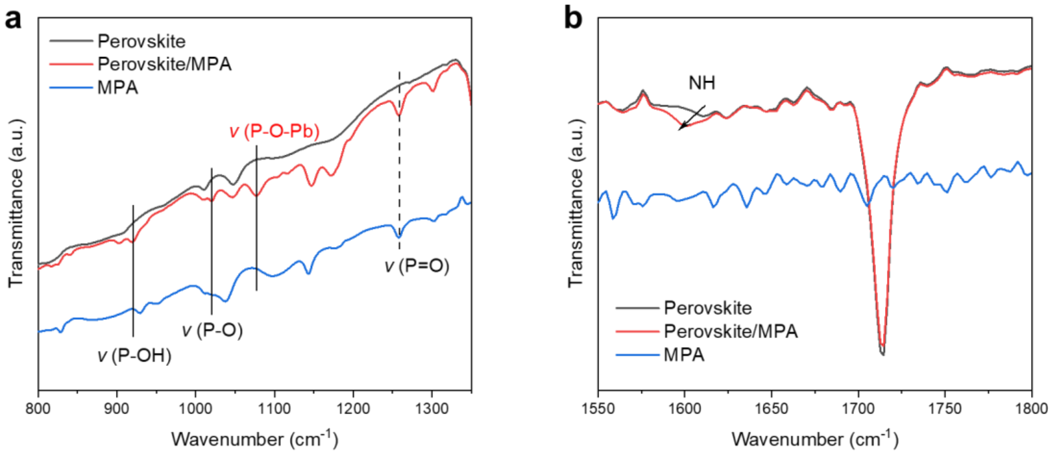

Supplementary Fig. 8. FTIR spectra of MPA, perovskite, MPA-modified perovskite films. The fingerprint regions highlight (a) P-OH, P-O, P=O stretching and (b) NH stretching.

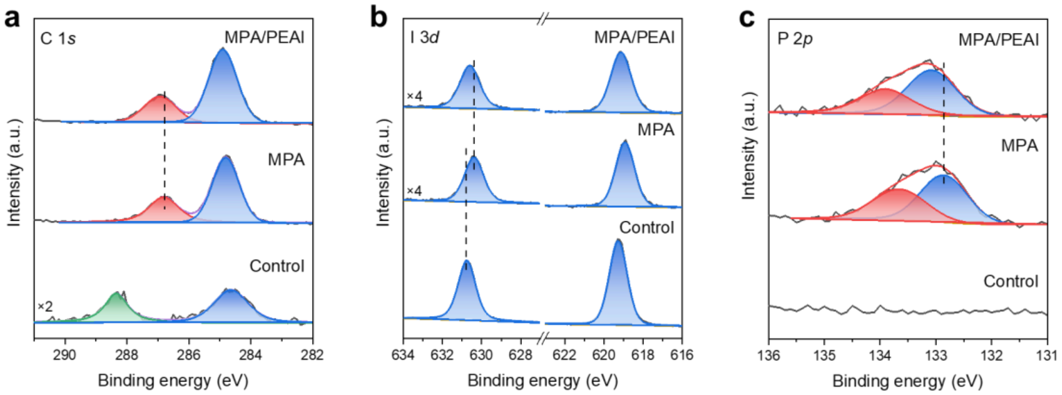

Supplementary Fig. 9. XPS measurement of perovskite films with SBI. XPS (a) C 1*s*, (b) I 3*d* and (c) P 2*p* core level spectra of control, MPA- and SBI-modified perovskite films.

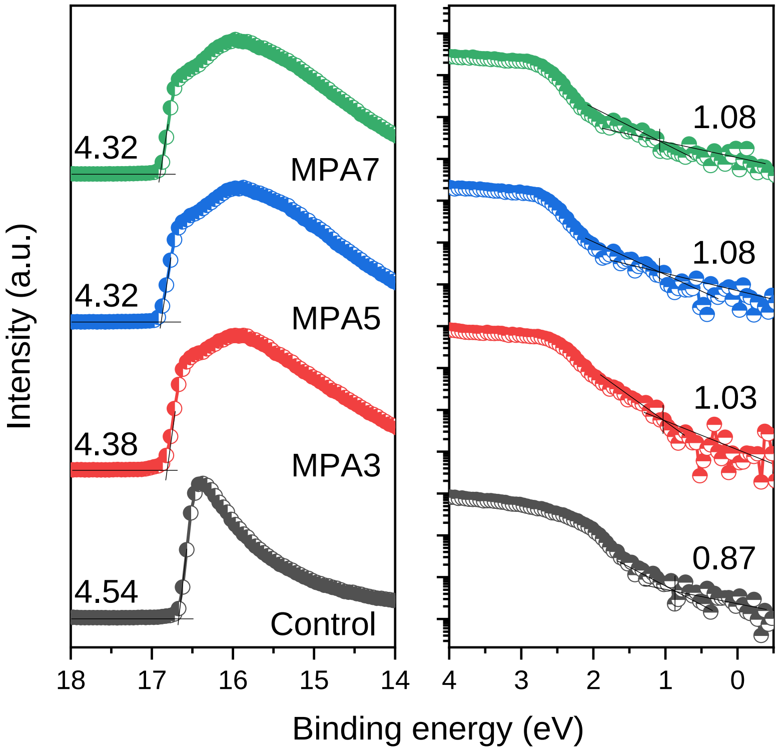

Supplementary Fig. 10. UPS spectra of secondary electron cutoff region and valence band region of control and MPA-modified perovskite films with different concentrations of 3, 5 and 7 mg ml^{-1}.

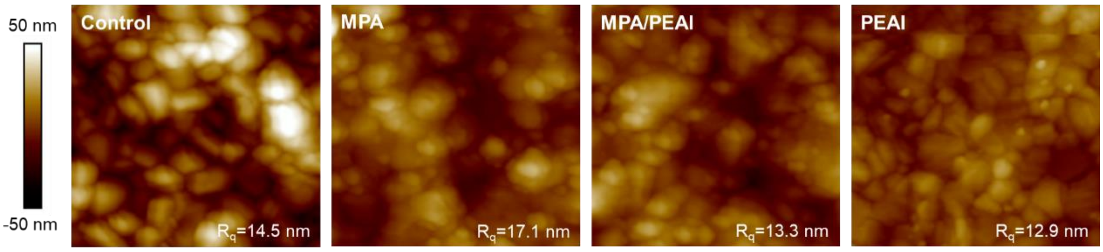

Supplementary Fig. 11. Topography images of control, MPA-, SBI- and PEAI-modified perovskite films.

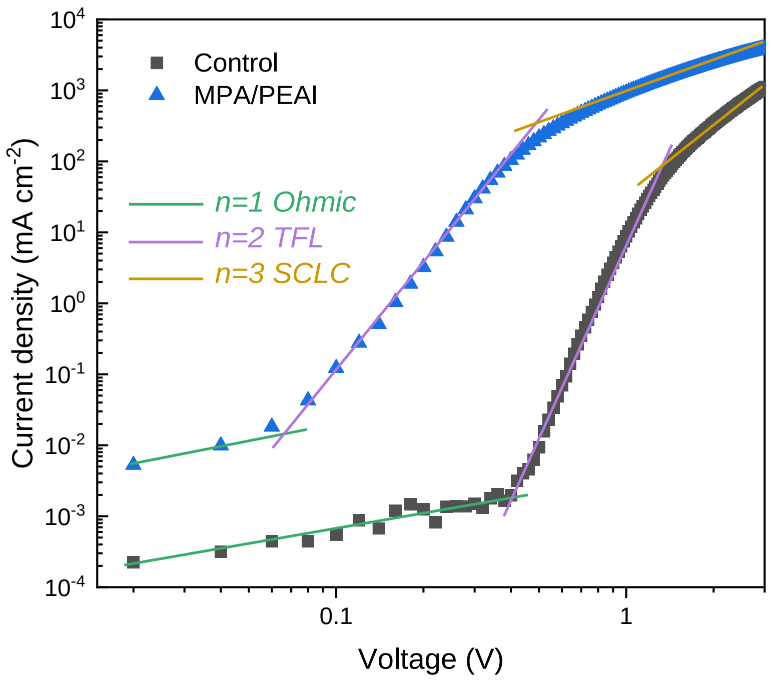

Supplementary Fig. 12. Dark *J-V* curves of electron-only devices with and without SBI modification.

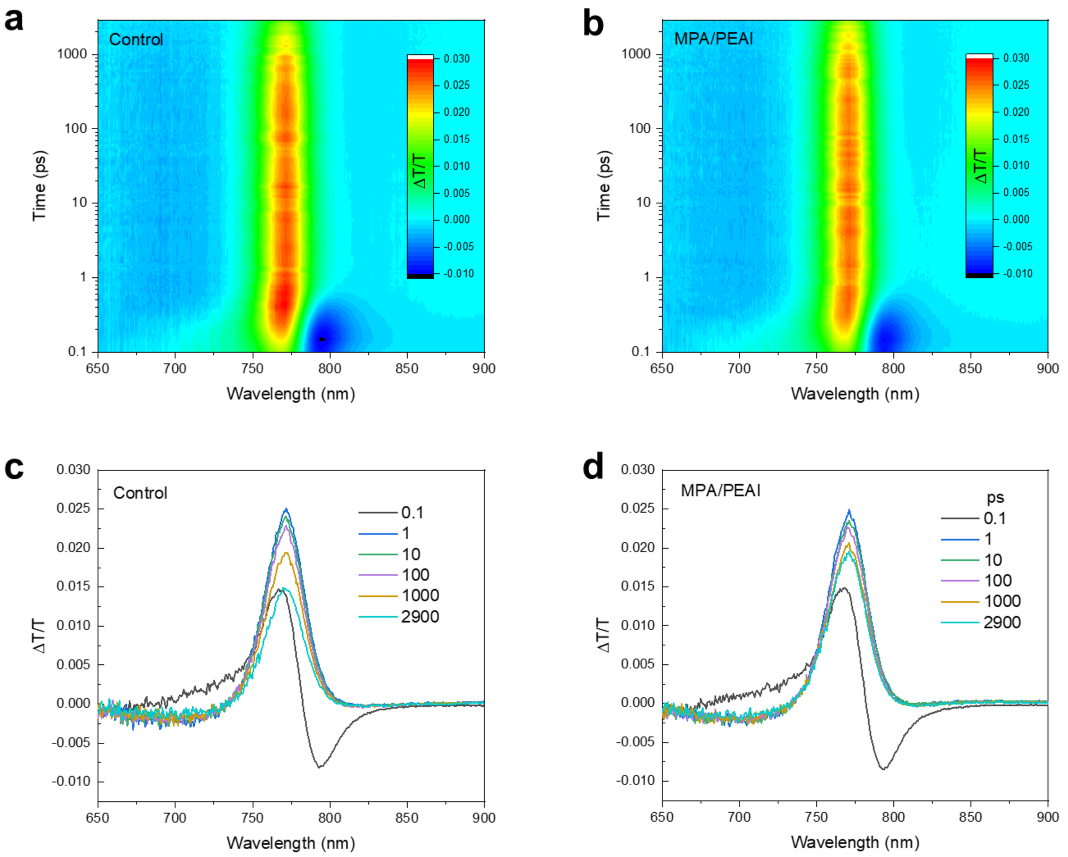

Supplementary Fig. 13. Impact of SBI on charge carrier dynamics. Two-dimensional (2D) pseudo-color plots of the fs-TA results for (a) control and (b) SBI-modified perovskite films. fs-TA spectra at selected pump-probe delay time of (c) control and (d) SBI-modified perovskite films.

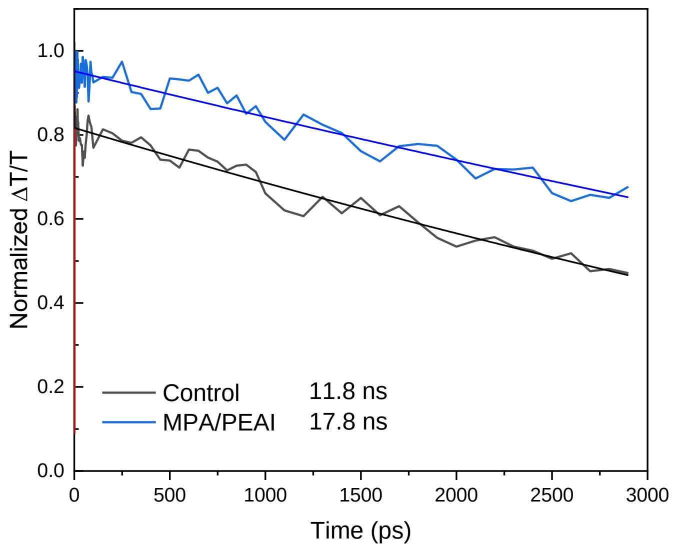

Supplementary Fig. 14. The GSB decays at 770 nm of control and SBI-modified perovskite films.

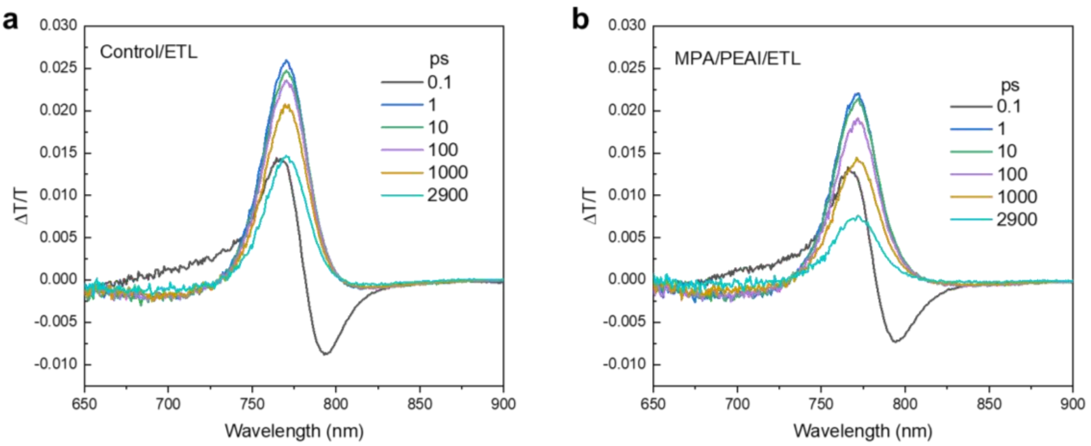

Supplementary Fig. 15. fs-TA spectra at selected pump-probe delay time of (a) control and (b) SBI-modified perovskite films with ETL.

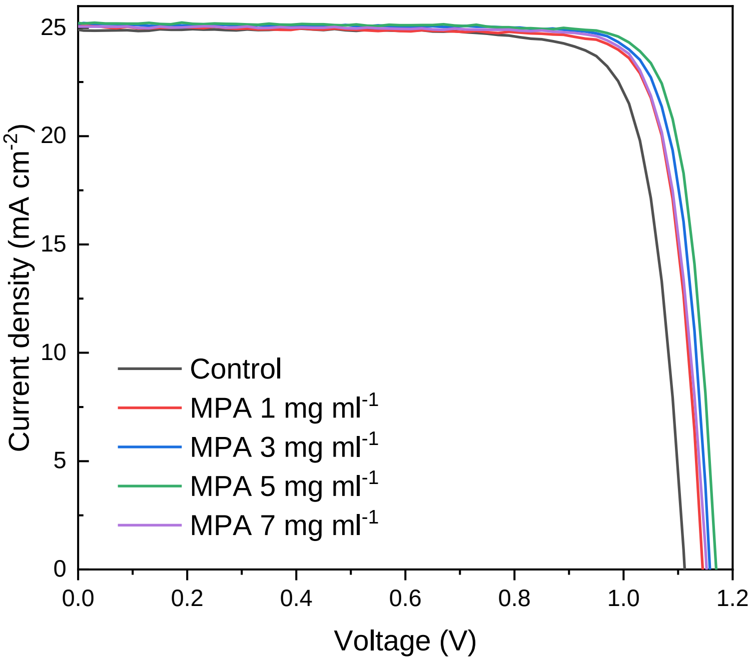

Supplementary Fig. 16. *J-V* curves of control and SBI-based PSCs with concentrations of 1, 3, 5 and 7 mg ml^{-1}. An optimal MPA concentration of 5 mg ml^{-1} is obtained.

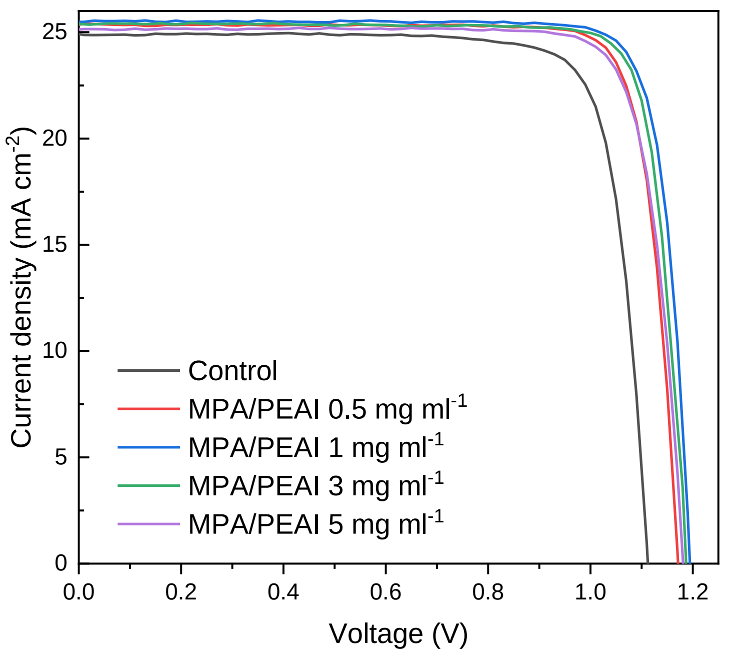

Supplementary Fig. 17. *J-V* curves of control and SBI-based PSCs with MPA concentration of 5 mg ml^{-1} and PEAI concentrations of 0.5, 1, 3 and 5 mg ml^{-1}. An optimal PEAI concentration of 1 mg ml^{-1} is obtained.

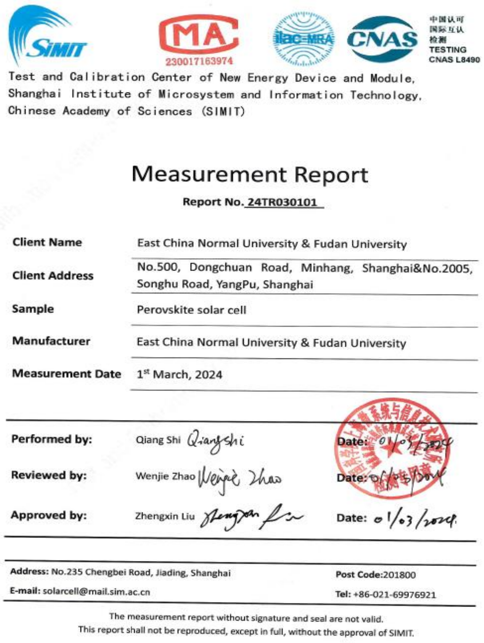

Test and Calibration Center of New Energy Device and Module,
Shanghai Institute of Microsystem and Information Technology,
Chinese Academy of Sciences (SIMIT)

230017163974

中国认可 国际互认 检测 TESTING CNAS L8490

Measurement Report

Report No. 24TR030101

**Client Name**	East China Normal University & Fudan University
**Client Address**	No.500, Dongchuan Road, Minhang, Shanghai&No.2005, Songhu Road, YangPu, Shanghai
**Sample**	Perovskite solar cell
**Manufacturer**	East China Normal University & Fudan University
**Measurement Date**	1st March, 2024

**Performed by:**	Qiang Shi *Qiangshi*	**Date:** 01/03/2024
**Reviewed by:**	Wenjie Zhao *Wenjie Zhao*	**Date:** 01/03/2024
**Approved by:**	Zhengxin Liu *Zhengxin Liu*	**Date:** 01/03/2024

Address: No.235 Chengbei Road, Jiading, Shanghai — **Post Code:** 201800
E-mail: solarcell@mail.sim.ac.cn — **Tel:** +86-021-69976921

The measurement report without signature and seal are not valid.
This report shall not be reproduced, except in full, without the approval of SIMIT.

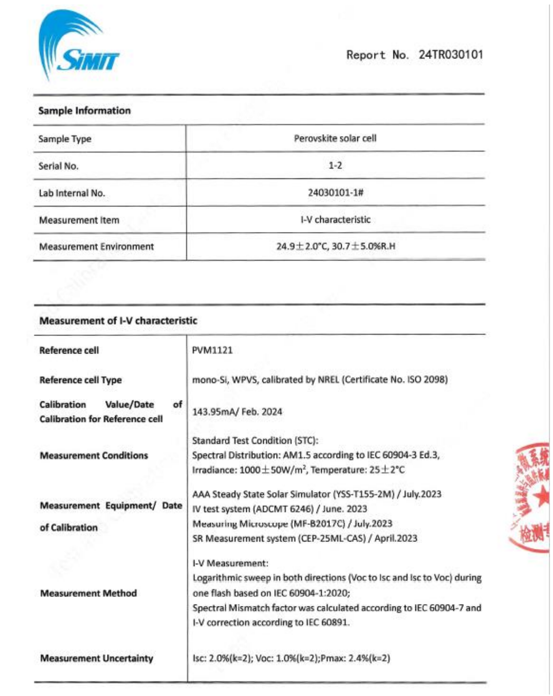

Report No. 24TR030101

**Sample Information**	
Sample Type	Perovskite solar cell
Serial No.	1-2
Lab Internal No.	24030101-1#
Measurement Item	I-V characteristic
Measurement Environment	24.9±2.0°C, 30.7±5.0%R.H

**Measurement of I-V characteristic**	
**Reference cell**	PVM1121
**Reference cell Type**	mono-Si, WPVS, calibrated by NREL (Certificate No. ISO 2098)
**Calibration Value/Date of Calibration for Reference cell**	143.95mA/ Feb. 2024
**Measurement Conditions**	Standard Test Condition (STC): Spectral Distribution: AM1.5 according to IEC 60904-3 Ed.3, Irradiance: 1000±50W/m², Temperature: 25±2°C
**Measurement Equipment/ Date of Calibration**	AAA Steady State Solar Simulator (YSS-T155-2M) / July.2023<br>IV test system (ADCMT 6246) / June. 2023<br>Measuring Microscope (MF-B2017C) / July.2023<br>SR Measurement system (CEP-25ML-CAS) / April.2023
**Measurement Method**	I-V Measurement:<br>Logarithmic sweep in both directions (Voc to Isc and Isc to Voc) during one flash based on IEC 60904-1:2020;<br>Spectral Mismatch factor was calculated according to IEC 60904-7 and I-V correction according to IEC 60891.
**Measurement Uncertainty**	Isc: 2.0%(k=2); Voc: 1.0%(k=2);Pmax: 2.4%(k=2)

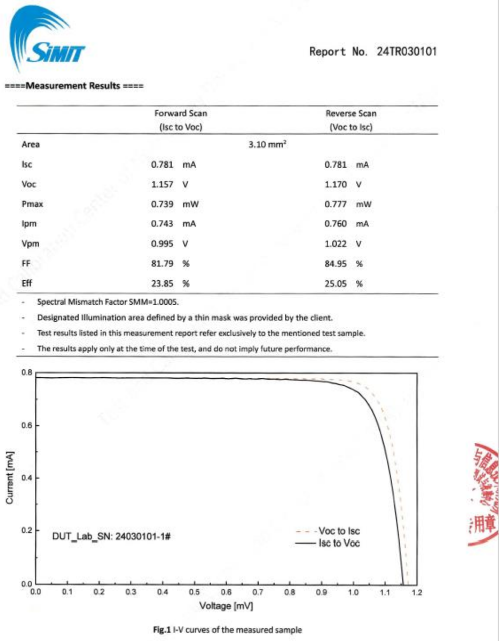

Report No. 24TR030101

====Measurement Results ====

	Forward Scan (Isc to Voc)		Reverse Scan (Voc to Isc)	
Area		3.10 mm²		
Isc	0.781	mA	0.781	mA
Voc	1.157	V	1.170	V
Pmax	0.739	mW	0.777	mW
Ipm	0.743	mA	0.760	mA
Vpm	0.995	V	1.022	V
FF	81.79	%	84.95	%
Eff	23.85	%	25.05	%

- Spectral Mismatch Factor SMM=1.0005.
- Designated Illumination area defined by a thin mask was provided by the client.
- Test results listed in this measurement report refer exclusively to the mentioned test sample.
- The results apply only at the time of the test, and do not imply future performance.

Fig.1 I-V curves of the measured sample

Supplementary Fig. 18. Device performance certification report by Shanghai Institute of Microsystem and Information Technology (SIMIT), Chinese Academy of Sciences. All elements in Supplementary Fig. 18 have received written approval from the copyright holder.

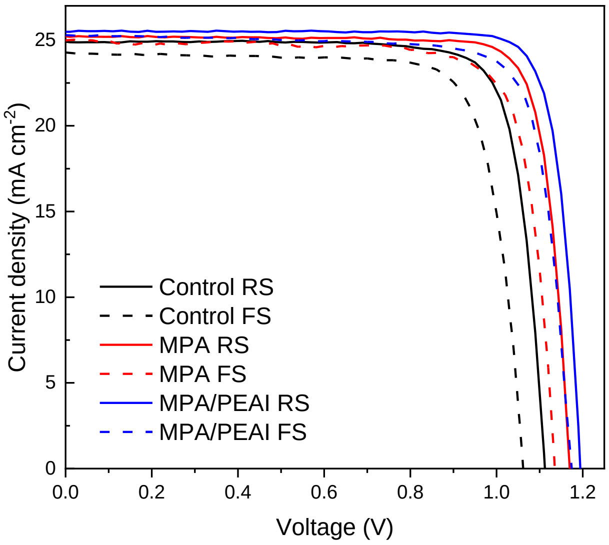

Supplementary Fig. 19. *J-V* curves of control, MPA- and SBI-based PSCs under forward scan (FS) and reverse scan (RS).

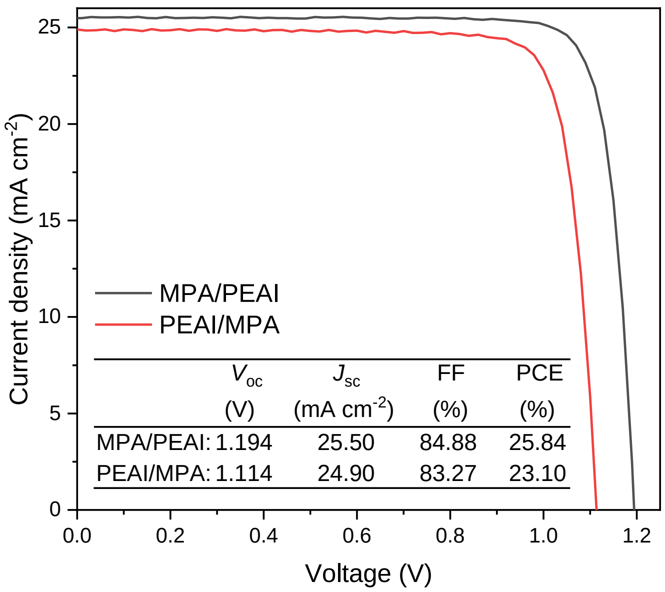

Supplementary Fig. 20. *J-V* curves of PSCs with MPA/PEAI and PEAI/MPA modification.

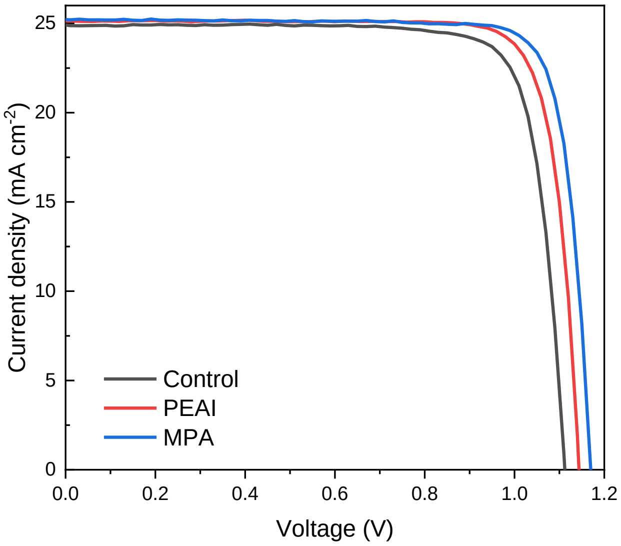

Supplementary Fig. 21. *J-V* curves of control, PEAI- and MPA-based PSCs.

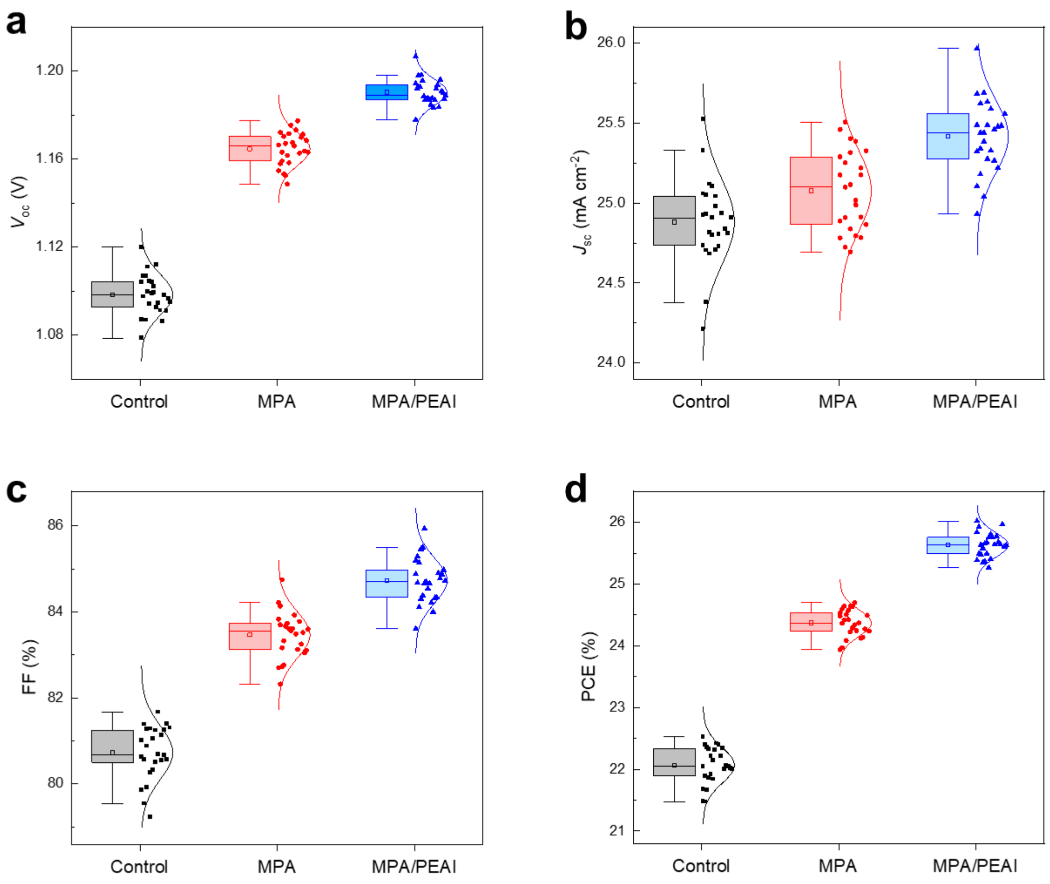

Supplementary Fig. 22. Statistics of photovoltaic parameters. (a) V_{oc}, (b) J_{sc}, (c) FF and (d) PCE obtained from 25 control, MPA- and SBI-based PSCs.

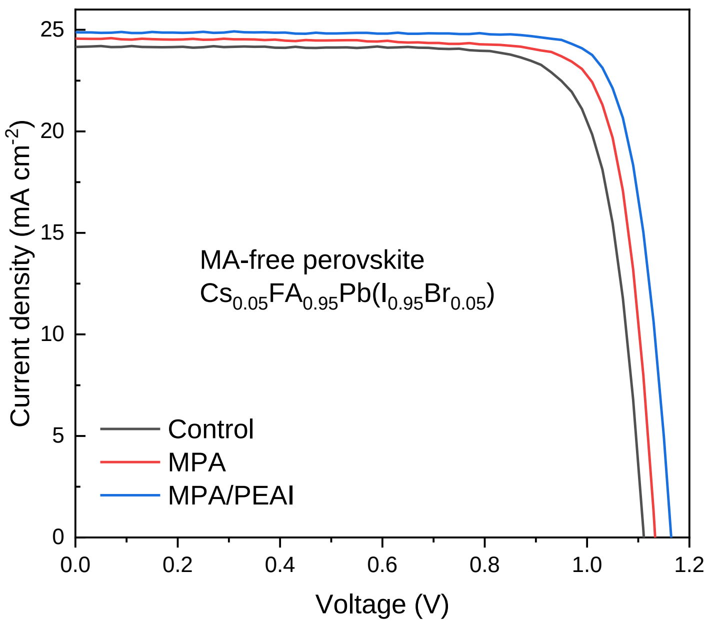

Supplementary Fig. 23. *J-V* curves of control, MPA- and SBI-based PSCs using MA-free perovskite $Cs_{0.05}FA_{0.95}Pb(I_{0.95}Br_{0.05})_3$.

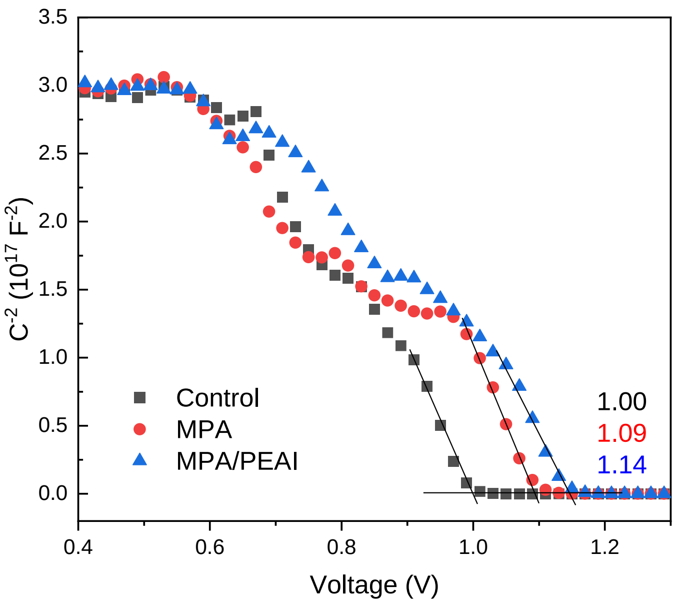

Supplementary Fig. 24. *C*-*V* curves of control, MPA- and SBI-based PSCs.

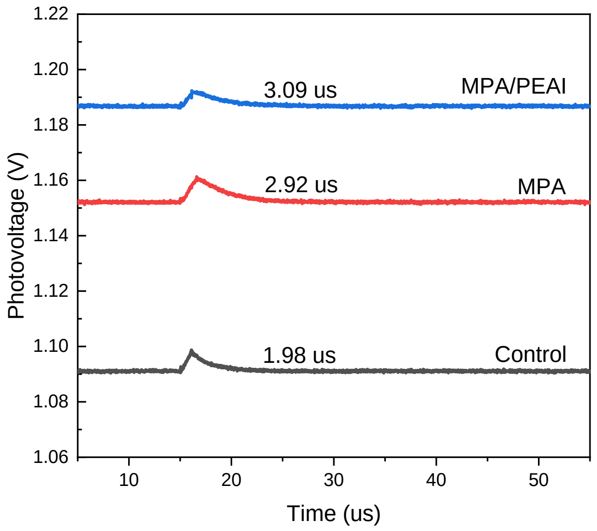

Supplementary Fig. 25. TPV decays of control, MPA- and SBI-based PSCs.

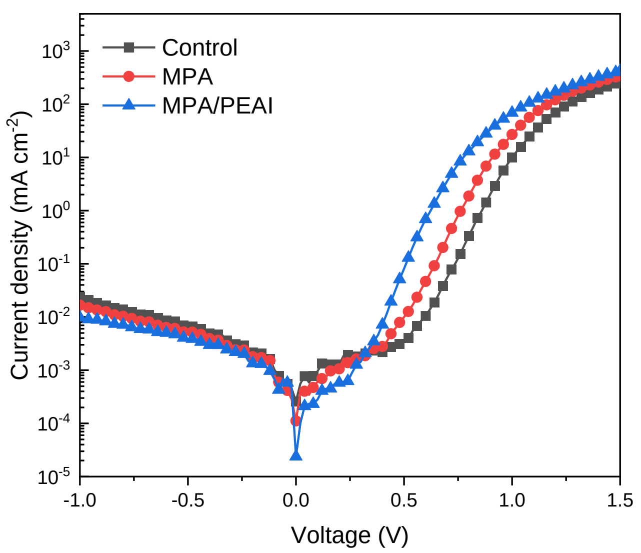

Supplementary Fig. 26. Dark *J-V* curves of control, MPA- and SBI-based PSCs.

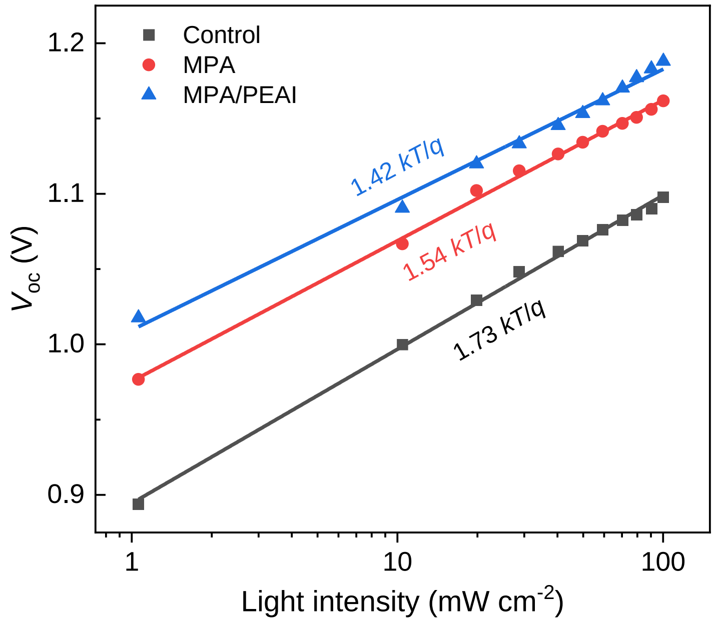

Supplementary Fig. 27. Light intensity dependent V_{oc} of control, MPA- and SBI-based PSCs.

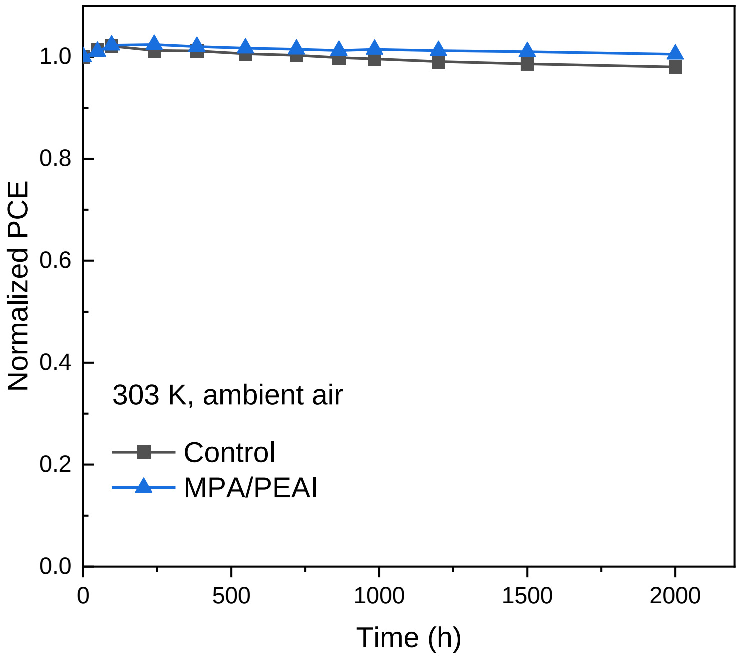

Supplementary Fig. 28. Normalized PCE evolution of control and SBI-based PSCs stored in ambient air at 303 K.

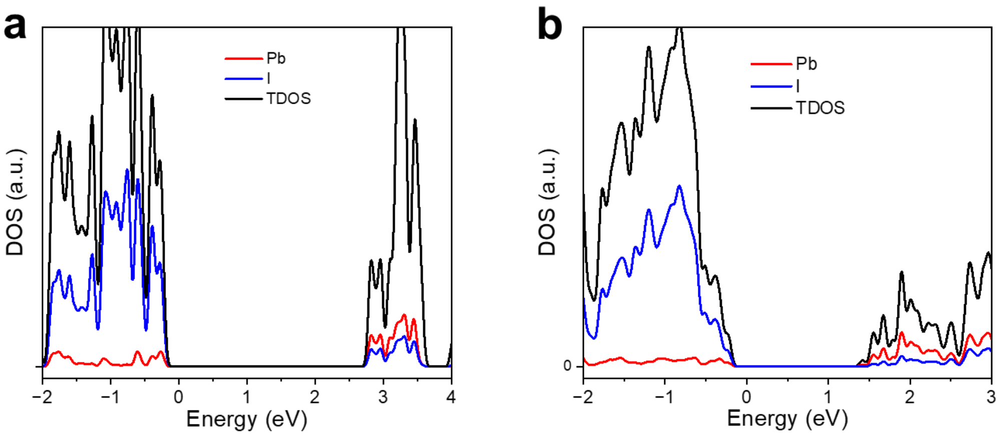

Supplementary Fig. 29. Density of states (DOS) plot of (a) FAI-terminated and (b) PbI-terminated perovskite (001) surface without iodine vacancy.

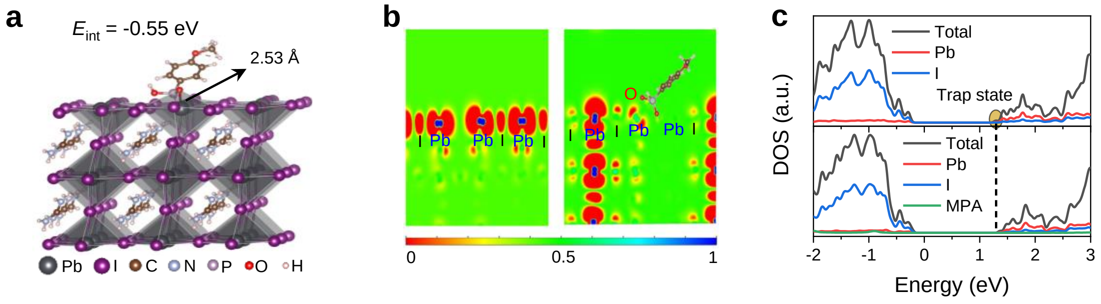

Supplementary Fig. 30. Theoretical analysis of interaction between perovskite and MPA. (a) Optimized structure of MPA treated PbI-terminated perovskite (001) surface containing an iodine vacancy. (b) Calculated electron localization function and (c) density of states (DOS) projected onto elements of PbI-terminated surface with an iodine vacancy before and after MPA treatment.

Supplementary Table 1. The fitted carrier lifetime of control and SBI-modified perovskite films with and without ETL obtained from the TRPL spectra (refer to Fig. 3c).

	**A_1**	**τ_1**	**A_2**	**τ_2**	**τ_{avg}**
Control	0.74	2.10	0.26	25.97	21.50
MPA/PEAI	0.58	5.65	0.42	62.76	56.45
Control (w ETL)	0.78	1.48	0.22	10.99	7.92
MPA/PEAI (w ETL)	0.68	0.95	0.32	5.36	4.15

Note: The TRPL decay is fitted by a bi-exponential equation: $y = A_1 \exp\left(-\frac{t}{\tau_1}\right) + A_2\, exp\left(-\frac{t}{\tau_2}\right)$, where parameters A_1 and A_2 are the amplitude fraction for each decay component, τ_1 and τ_2 represent the time constant of the two types of decay. The average lifetime (τ_{avg}) can be calculated with the equation: $\tau_{avg} = \frac{(A_1\tau_1^2+A_2\tau_2^2)}{(A_1\tau_1+A_2\tau_2)}$.

Supplementary Table 2. Photovoltaic parameters extracted from *J-V* curves of control and MPA-modified devices with concentrations of 1, 3, 5 and 7 mg ml^{-1} (refer to Supplementary Figure 16).

	V_{oc} [V]	J_{sc} [mA cm^{-2}]	FF [%]	PCE [%]
Control	1.112	24.91	81.31	22.52
MPA 1	1.145	25.09	82.98	23.84
MPA 3	1.158	25.10	83.83	24.36
MPA 5	1.170	25.22	83.52	24.64
MPA 7	1.152	25.05	83.25	24.03

Supplementary Table 3. Photovoltaic parameters extracted from *J*-*V* curves of control and MPA-based devices with MPA concentration of 5 mg ml^{-1} and PEAI concentrations of 0.5, 1, 3 and 5 mg ml^{-1} (refer to Supplementary Figure 17).

	V_{oc} [V]	J_{sc} [mA cm^{-2}]	FF [%]	PCE [%]
Control	1.112	24.91	81.31	22.52
MPA/PEAI 0.5	1.171	25.36	84.19	25.00
MPA/PEAI 1	1.194	25.50	84.88	25.84
MPA/PEAI 3	1.187	25.39	84.47	25.46
MPA/PEAI 5	1.181	25.12	83.05	24.64

Supplementary Table 4. Photovoltaic parameters extracted from *J-V* curves of control and SBI-based devices (refer to Fig. 4a, Supplementary Figure 22).

	**V_{oc}** **[V]**	**J_{sc}** **[mA cm^{-2}]**	**FF** **[%]**	**PCE** **[%]**
Control	1.098 ± 0.009 (1.112)	24.88 ± 0.29 (24.91)	80.73 ± 0.61 (81.98)	22.06 ± 0.26 (22.52)
MPA	1.165 ± 0.007 (1.170)	25.08 ± 0.21 (25.22)	83.47 ± 0.52 (83.52)	24.37 ± 0.24 (24.64)
MPA/PEAI	1.190 ± 0.006 (1.194)	25.42 ± 0.22 (25.50)	84.73 ± 0.51 (84.88)	25.63 ± 0.19 (25.84)

Note: The parameters listed in parentheses are the best performing devices (refer to Fig. 4a). The plus-minus sign (±) represents the standard deviation. Perovskite with bandgap of 1.55 eV is employed in this work, which corresponds to a theoretical PCE of 31.02% and J_{sc} of 27.26 mA cm^{-2} according to the S-Q limit.[8,9]

Supplementary Table 5. Photovoltaic parameters extracted from *J-V* curves of control, PEAI- and MPA-based PSCs. (refer to Supplementary Figure 21).

	V_{oc} [V]	J_{sc} [mA cm^{-2}]	FF [%]	PCE [%]
Control	1.112	24.91	81.31	22.52
PEAI	1.144	25.13	82.97	23.85
MPA	1.170	25.22	83.52	24.64

Supplementary Table 6. Photovoltaic parameters extracted from *J-V* curves of control and SBI-based devices based on MA-free perovskite $Cs_{0.05}FA_{0.95}Pb(I_{0.95}Br_{0.05})_3$ (refer to Supplementary Figure 23).

	V_{oc} [V]	J_{sc} [mA cm^{-2}]	FF [%]	PCE [%]
Control	1.111	24.16	79.59	21.36
MPA	1.133	24.57	82.05	22.84
MPA/PEAI	1.164	24.88	82.84	23.99

Supplementary Table 7. $\Delta V_{oc,\ nonrad}$ and V_{oc} of recent works on p-i-n PSCs (refer to Figure 4f).

$\Delta V_{oc,\ nonrad}$ [mV]	V_{oc} [V]	
170	1.126	ref 1
60.13	1.18	ref 2
113.91	1.16	ref 3
68.75	1.184	ref 4
71	1.18	ref 5
76.86	1.176	ref 6
**59**	**1.194**	**This work**

Supplementary Note 1. Estimated thickness of the MPA and PEAI by the intensity attenuation of Pb 4*f* XPS spectra.

Specifically, the thickness of the MPA and PEAI are calculated to be about 2.21 and 2.37 nm, respectively, according to the intensity attenuation of Pb 4*f* XPS spectra by the equation[7]: $d = \lambda \cos\theta \ln\left(\frac{I}{I_0} + 1\right)$, where λ the inelastic mean free path for Pb 4*f* photoelectrons, θ is the emission angle of the photoelectron with respect to the sample surface normal, I_0 and I are peak intensity of Pb 4f before and after treatment (Fig. 1d).

Supplementary References

1 Liu, K. *et al.* Zwitterionic-surfactant-assisted room-temperature coating of efficient perovskite solar cells. *Joule* **4**, 2404-2425 (2020).

2 Li, F. *et al.* Regulating surface termination for efficient inverted perovskite solar cells with greater than 23% efficiency. *J. Am. Chem. Soc.* **142**, 20134-20142 (2020).

3 Sun, X. *et al.* Efficient inverted perovskite solar cells with low voltage loss achieved by a pyridine-based dopant-free polymer semiconductor. *Angew. Chem., Int. Ed.* **60**, 7227-7233 (2021).

4 Li, Z. *et al.* Organometallic-functionalized interfaces for highly efficient inverted perovskite solar cells. *Science* **376**, 416-420 (2022).

5 Niu, B. *et al.* Multifunctional hybrid interfacial layers for high-performance inverted perovskite solar cells. *Adv. Mater.* **35**, 2212258 (2023).

6 Chen, R. *et al.* Reduction of bulk and surface defects in inverted methylammonium- and bromide-free formamidinium perovskite solar cells. *Nat. Energy* **8**, 839-849 (2023).

7 Iwai, H., Hammond, J. S. & Tanuma, S. Recent status of thin film analyses by XPS. *J. Surf. Anal.* **15**, 264-270 (2009).

8 Ma, C. & Park, N.-G. A realistic methodology for 30% efficient perovskite solar cells. *Chem* **6**, 1254-1264 (2020).

9 Rühle, S. Tabulated values of the Shockley-Queisser limit for single junction solar cells. *Sol. Energy* **130**, 139-147 (2016)
