# Supplementary material for: Reducing nonradiative recombination for highly efficient inverted perovskite solar cells via a synergistic bimolecular interface
Source: Nat Commun. 2024 Jul 4;15:5607. doi: 10.1038/s41467-024-50019-3 (PMC11224317; doi:10.1038/s41467-024-50019-3)
Supplement: Supplementary file 3 — Reporting Summary [file 41467_2024_50019_MOESM3_ESM.pdf]

nature portfolio

Corresponding author(s): Lijun Zhang, Feng Gao, Qinye Bao

Solar Cells Reporting Summary

Nature Portfolio wishes to improve the reproducibility of the work that we publish. This form is intended for publication with all accepted papers reporting the characterization of photovoltaic devices and provides structure for consistency and transparency in reporting. Some list items might not apply to an individual manuscript, but all fields must be completed for clarity.

For further information on Nature Research policies, including our data availability policy, see Authors & Referees.

Experimental design

Please check the following details are reported in the manuscript, and provide a brief description or explanation where applicable.

1. Dimensions

Area of the tested solar cells

- [x] Yes — The area of tested device is 0.05 cm-2.
- [ ] No — *Explain why this information is not reported/not relevant.*

Method used to determine the device area

- [x] Yes — A metal shadow mask with the area of 0.05 cm-2 is used during metal electrode deposition.
- [ ] No — *Explain why this information is not reported/not relevant.*

2. Current-voltage characterization

Current density-voltage (J-V) plots in both forward and backward direction

- [x] Yes — Supplementary Figure 19
- [ ] No

Voltage scan conditions

- [x] Yes — "Device characterization" of "Method"
- [ ] No — *Explain why this information is not reported/not relevant.*

Test environment

- [x] Yes — "Device characterization" of "Method"
- [ ] No — *Explain why this information is not reported/not relevant.*

Protocol for preconditioning of the device before its characterization

- [ ] Yes — *Provide a description of the protocol.*
- [x] No — Without any preconditioning

Stability of the J-V characteristic

- [x] Yes — Figure 4c and 4i, Supplementary Figure 28
- [ ] No — *Explain why this information is not reported/not relevant.*

3. Hysteresis or any other unusual behaviour

Description of the unusual behaviour observed during the characterization

- [x] Yes — Section of "Photovoltaic performance and energy loss"
- [ ] No — *Explain why this information is not reported/not relevant.*

Related experimental data

- [x] Yes — Supplementary Figure 19
- [ ] No — *Explain why this information is not reported/not relevant.*

4. Efficiency

External quantum efficiency (EQE) or incident photons to current efficiency (IPCE)

- [x] Yes — Figure 4b
- [ ] No — *Explain why this information is not reported/not relevant.*

A comparison between the integrated response under the standard reference spectrum and the response measure under the simulator

- [x] Yes — The integrated Jsc values from EQE are consistent with Jsc values from J-V curves.
- [ ] No — *Explain why this information is not reported/not relevant.*

For tandem solar cells, the bias illumination and bias voltage used for each subcell

- [ ] Yes
- [x] No

Provide a description of the measurement conditions.

No tandem solar cells reported.

5. Calibration

Light source and reference cell or sensor used for the characterization

- [x] Yes
- [ ] No

Solar simulator (SS-F5-3A, Enlitech) is used for the characterization. "Device characterization" of "Method"

Explain why this information is not reported/not relevant.

Confirmation that the reference cell was calibrated and certified

- [x] Yes
- [ ] No

The light intensity (AM 1.5G, 100 mW cm-2) was calibrated with a NREL-certified Si cells. "Device characterization" of "Method"

Explain why this information is not reported/not relevant.

Calculation of spectral mismatch between the reference cell and the devices under test

- [ ] Yes
- [x] No

Provide a value of the spectral mismatch and/or a description of how it has been taken into account in the measurements.

We did not calculate the spectral mismatch between the reference cell and the devices under test.

6. Mask/aperture

Size of the mask/aperture used during testing

- [x] Yes
- [ ] No

A metal aperture mask with the area of 0.031 cm-2 is used for testing.

Explain why this information is not reported/not relevant.

Variation of the measured short-circuit current density with the mask/aperture area

- [ ] Yes
- [x] No

Report the difference in the short-circuit current density values measured with the mask and aperture area.

All devices are measured with the same metal aperture mask.

7. Performance certification

Identity of the independent certification laboratory that confirmed the photovoltaic performance

- [x] Yes
- [ ] No

No record efficiency is claimed. However, the performance certification is also provided in Supplementary Figure 18.

Explain why this information is not reported/not relevant.

A copy of any certificate(s)

- [x] Yes
- [ ] No

Supplementary Figure 18

Explain why this information is not reported/not relevant.

8. Statistics

Number of solar cells tested

- [x] Yes
- [ ] No

Statistical analysis is performed based on 25 devices of each type.

Explain why this information is not reported/not relevant.

Statistical analysis of the device performance

- [x] Yes
- [ ] No

Figure 4d, Supplementary Figure 22

Explain why this information is not reported/not relevant.

9. Long-term stability analysis

Type of analysis, bias conditions and environmental conditions

- [x] Yes
- [ ] No

Figure 4c and 4i, Supplementary Figure 28

Explain why this information is not reported/not relevant.
